# The Treatment Selection for a 36-Year-Old Woman With Stress Urinary Incontinence Using a Discrete Mathematical Approach: A Case Report

**DOI:** 10.7759/cureus.61314

**Published:** 2024-05-29

**Authors:** Nobuo Okui

**Affiliations:** 1 Dentistry, Kanagawa Dental University, Yokosuka, JPN

**Keywords:** personalized medicine, vaginal erbium laser, graph theory, overactive bladder, stress urinary incontinence, discrete mathematics

## Abstract

This case report describes the treatment selection process for a 36-year-old woman with stress urinary incontinence (SUI) and an overactive bladder (OAB) who desired pregnancy. The patient had comorbidities of hypertension and type 2 diabetes, which required consideration to improve her quality of life and reproductive health. A recently developed decision support tool using a discrete mathematical approach was used to select a treatment method tailored to the patient's individual situation. The analysis determined that vaginal erbium laser (VEL) treatment (Renovalase SP Dynamis Fotona d.o.o, Ljubljana, Slovenia) was the most suitable for this patient. VEL treatment significantly improved both SUI and OAB and changing antihypertensive medication eliminated nocturia. This case suggests the potential application of graph theory in treatment selection for SUI patients.

## Introduction

Stress urinary incontinence (SUI) and overactive bladder (OAB) are common urological conditions that significantly impair women's quality of life [1]. SUI refers to involuntary leakage of urine during increased abdominal pressure, such as coughing, sneezing, or physical activity. OAB is a syndrome characterized by urgency, frequency, and urge incontinence [2]. These conditions have a substantial impact on the patient's quality of life, including limitations in social activities, sleep disturbances, and decreased sexual function [3].

Treatment options for SUI range from conservative management to surgical intervention. Conservative management includes pelvic floor muscle training, behavioral therapy, and pharmacotherapy [4]. The current gold standard for surgical treatment is mid-urethral sling surgery (MUS) [5]. Tension-free vaginal tape (TVT), a representative MUS technique, has been reported to have high efficacy and low complication rates [5]. TVT is an invasive surgery with risks of complications such as postoperative pain and voiding dysfunction [6,7]. Japanese guidelines recommend MUS for patients with simultaneous SUI and OAB based on evidence [8-10]. However, surgical targets and criteria have not been clarified [8]. Vaginal erbium laser (VEL) treatment has gained attention as a minimally invasive treatment option [11,12]. VEL uses laser technology to promote collagen regeneration in the vaginal and urethral mucosa, thereby improving SUI and OAB symptoms [11,12]. VEL is noninvasive, has a low risk of complications, and is applicable to patients who desire pregnancy [2].

The selection of treatment for SUI and OAB requires consideration of various factors, such as the patient's age, severity, comorbidities, and desire for pregnancy [2]. While each country has established guidelines for the treatment of SUI based on the best available evidence, these guidelines do not cover all aspects of medical decision-making [8,13-20]. All guidelines serve as tools for clinicians to make informed decisions about patient care, presenting various types of information, such as the importance of post-treatment follow-up and patient education. Treatment options for stress urinary incontinence include conservative management, mid-urethral slings (transobturator and retropubic approaches), single-incision slings, urethral bulking agents, and stem cell therapy. Furthermore, future directions will discuss new treatment options, such as laser therapy and magnetic/electrical stimulation therapies, as well as the need for standardization of outcome assessments. However, to ensure that all physicians use this information in a balanced manner, ideas supporting these guidelines are necessary. Considering the individual circumstances and values of patients is crucial for patient-centered care. Exploring new approaches and research is essential for the advancement of medicine, and may provide patients with new treatment options.

Recently, decision support tools using graph theory have been developed and utilized to address the difficulty of comprehensively evaluating these factors and selecting the optimal treatment [21]. Graph theory is a branch of discrete mathematics that analyzes network structures and is used to analyze complex discrete data [22]. It has been adopted in most modern complex discrete models, such as Google Maps, air traffic control models, and Amazon delivery schedules [22]. Using graph theory, patient conditions and treatment characteristics can be modeled as variables and the optimal treatment can be derived [21]. The use of discrete mathematics may play a complementary role in utilizing the guidelines constructed for SUI and may strengthen physicians' explanations [21].

This case report describes the process of utilizing a decision support tool based on graph theory to select a treatment method tailored to the individual situation of a 36-year-old woman with both SUI and OAB, who desired pregnancy. This case suggests the potential application of graph theory in treatment selection for SUI patients and contributes to the practice of personalized medicine.

## Case presentation

A 36-year-old woman (height: 159 cm; weight: 71 kg; body mass index: 28.08 kg/m^2^) presented with a history of urinary incontinence since her first delivery at the age of 23. Her condition worsened over time, particularly in the last three years after the development of hypertension and type 2 diabetes. At worst, she experienced leakage of 100 ml per hour during physical activity. A 1-hour pad test showed 32 g of leakage, and her overactive bladder symptom score (OABSS) was 7 points. Her medical history included hypertension (145/92 mmHg, treated with amlodipine 5 mg once daily), type 2 diabetes (HbA1c 6.9%, treated with dapagliflozin 5 mg once daily), and cellulitis of the left lower limb. The patient desired pregnancy and required consideration of both QOL improvement and reproduction. The patient frequently developed bacterial cystitis. The angle change in the bladder neck was 32° using the Valsalva method. During reproductive medical treatment, the use of ovulation inducers (Clomid, Serophene) increased the frequency of urinary urgency. According to Japanese SUI guidelines, urodynamic studies are not considered necessary for the diagnosis of SUI, and decisions are made based on the aforementioned criteria. Additionally, for OAB, only questionnaires were used for assessment [8]. Currently, she is receiving supplementary therapy with vitamin D, folic acid, zinc, and omega-3 fatty acids.

Informed consent process at other hospitals

Doctor A recommended TVT, citing high improvement rates in the 1-hour pad test. Explained the possibility of pharmacotherapy, botulinum bladder injection, or sacral neuromodulation (SNM) if OAB worsened after TVT. No explanation was provided regarding the desire for pregnancy.

Doctor B explained TVT at a reproduction clinic. The impact of surgery on reproduction was unclear, and reproduction should be prioritized.

Doctor C pointed out the possibility of invasive SUI treatment inducing cellulitis, considering the patient's comorbidities.

Doctor D recommended transobturator tape (TOT); TOT has a smaller insertion range than TVT, reducing the risk of venous mispuncture due to obesity. Proposed OAB medications and botulinum bladder injections.

As the opinions of multiple doctors were inconsistent, the patient visited our hospital's urinary incontinence specialty outpatient clinic. The patient stated that she could not prioritize the improvement of SUI and OAB, desire for pregnancy, hypertension, or diabetes.

Treatment policy decisions at our hospital

At our hospital, we propose TVT and TOT as outpatient surgeries, as recommended by the Japanese guidelines for lower urinary tract symptoms, with a monthly record of 4-12 cases. However, urethral submucosal collagen injection, as indicated in the guidelines, has not been approved and cannot be performed in the patient's area of residence. We offer VEL as an alternative to TVT, with a monthly record of 30-40 cases. Botulinum toxin A bladder mucosal injection, recommended by the OAB guidelines, is performed 15-20 times per week. Pelvic organ prolapse surgery was performed 15-25 times per week. All patients can select all options without bias.

External findings of the urethra revealed dryness of the entire vagina, which was more than expected for her age. This can be attributed to hormonal changes that affect both the urethra and vagina, as noted in the literature [23-25]. The urethral hypermobility was mild. No pelvic organ prolapse such as cystocele or uterine prolapse was observed. Urine cytology and cervical smear cytology were normal.

Table 1 shows the results of blood tests. There was no anemia, and the kidney and liver functions were normal, indicating that TVT surgery was possible. However, the HbA1c value raises concerns regarding postoperative and polypropylene mesh infections. There is a risk of cellulitis from the insertion site to the thigh in TOT surgery. Dapagliflozin has been chosen to prevent further obesity progression in diabetes treatment, but it is also one of the causes of urinary tract infections. SUI causes repeated infections between the pad and skin, so treatment of SUI is necessary to reduce the possibility of infection, which hinders exercise. In contrast, urinary tract infections increase the risk of infection in TVT and TOT patients. Although there is no direct evidence that Clomid and Serophene worsen OAB, there are several aspects of the possibility that hormonal changes caused by clomiphene may be associated with OAB and urinary symptoms. Clomiphene, a selective estrogen receptor modulator (SERM), blocks estrogen receptors in the pituitary gland and increases the secretion of gonadotropins (LH and FSH), resulting in the promotion of follicle development and increased estrogen production. The fluctuation of estrogen levels may affect the bladder mucosa and muscles, possibly increasing urinary urgency and frequency. Amlodipine may cause nocturnal polyuria and worsen OABSS by inducing peripheral vasodilation and edema (especially around the ankles). Daytime edema may be redistributed when lying down at night, leading to increased urine volume, which is nocturnal polyuria. However, it was difficult to make a definitive judgment at this point.

**Table 1:** Initial blood test results Measured values and normal ranges. PT time: prothrombin time; PTT activity: partial thromboplastin time activity; PT-INR: prothrombin time - international normalized ratio; WBC: white blood cell count; RBC: red blood cell count; Hb: hemoglobin; Ht: hematocrit; MCV: mean corpuscular volume; MCH: mean corpuscular hemoglobin; MCHC: mean corpuscular hemoglobin concentration; APTT: activated partial thromboplastin time; TP: total protein (coagulation factor); AST (GOT): aspartate aminotransferase (glutamate-oxaloacetate transaminase); ALT (GPT): alanine aminotransferase (glutamate-pyruvate transaminase); LDH/FCC: lactate dehydrogenase/fully certified concentration; ALP/1FCC: alkaline phosphatase/fully certified concentration; γ-GT (γ-GTP): gamma-glutamyl transferase (gamma-glutamyl transpeptidase); CRE: creatinine; UA: urea acid; UN: urea nitrogen; HbA1c (NGSP): hemoglobin A1c (National Glycohemoglobin Standardization Program); TG: triglycerides; total cholesterol: total cholesterol; HDL cholesterol: high-density lipoprotein cholesterol; T-Bil: transaminogram (total bilirubin); eGFR: estimated glomerular filtration rate

Item	Result	Normal range
Prothrombin time (PT Time)	11.9 sec	10.0-13.0 sec
PTT activity	91.0 sec	80.0-120.0 sec
PT-INR	1.04	0.90-1.13
White blood cell count (WBC)	4.9x10^3/μL	3.5-9.7x10^3/μL
Red blood cell count (RBC)	4.79x10^6/μL	3.76-5.16x10^6/μL
Hemoglobin (Hb)	13.2 g/dL	11.2-15.2 g/dL
Hematocrit (Ht)	38.2%	34.3-45.2%
MCV	80 fL	80-101 fL
MCH	27.5 pg	26.4-34.3 pg
MCHC	32.5%	31.3-36.1%
APTT	33.2 sec	26.0-38.0 sec
Coagulation factor (TP)	7.2 g/dL	6.5-8.2 g/dL
AST (GOT)	22 U/L	10-40 U/L
ALT (GPT)	23 U/L	5-45 U/L
LDH/FCC	158 U/L	120-245 U/L
ALP/1FCC	95 U/L	38-113 U/L
γ-GT (γ-GTP)	22 U/L	0-48 U/L
Creatinine (CRE)	0.34 mg/dL	0.46-0.82 mg/dL
Urea (UA)	4.4 mg/dL	2.7-7.0 mg/dL
Uric acid (UN)	9.6 mg/dL	8.0-20.0 mg/dL
HbA1c (NGSP)	6.9%	4.6-6.2%
Triglycerides (TG)	78 mg/dL	50-149 mg/dL
Total cholesterol	116 mg/dL	150-219 mg/dL
HDL cholesterol	40 mg/dL	40-90 mg/dL
Transaminogram (T-Bil)	0.7 mg/dL	0.1-1.0 mg/dL
eGFR	166.9 mL/min	60 ml/min/1.73 m^2^ or more

For reference, the results of the urodynamic examination are presented in Table 2. Although this examination is not mandatory according to guidelines, it provides useful information for understanding the patient's condition. However, it should be noted that the main focus of this case report is on the treatment selection process based on the patient's individual circumstances, and the urodynamic examination is only a part of this process.

Table 2 shows the bladder findings from a urodynamic examination using the Goby Urodynamic System (EDAP TMS Co., Tokyo, Japan). As the patient suffered from SUI, frequent urine leakage led to an earlier onset of initial urinary desire, with the first desire measured at 34.7 ml. Additionally, the habit of storing urine in the bladder diminished, resulting in a maximum cystometric capacity of 273.8 ml.

**Table 2:** Bladder findings from urodynamic examination using goby urodynamic system FD: first desire to void; ND: normal desire to void; MCC: maximum cystometric capacity; PV: permission to void; Pves: vesical pressure; Pabd: abdominal pressure; Pura: urethral pressure; Pdet: detrusor pressure

Time	Event name	Pves	Pabd	Pura	Pdet	Volume	EMG	Other
1.1	Start infusion	36.8	36.3	26.4	0.5	0	0.5	-15.7
27.2	FD first desire	34.7	33.9	22.7	0.7	0	-0.6	50
02:46.9	ND normal desire	32.6	32.6	23.7	0	0	0.8	50
02:50.5	Cough	48.6	46.1	25.3	2.5	0	0.5	50
02:53.8	Cough	73.3	73.5	27.6	3	0	0	50
02:56.8	Cough	60.3	60.2	29.7	2	0	0	50
04:40.6	MCC max capacity	34	34	25.1	0	0	0	50
04:41.2	PV permission to void	33.7	33.9	24.8	-0.2	0	0	50
06:03.0	Cough	70.6	64.9	27.9	5.6	0	0	50
06:05.3	Cough	78.1	74.4	27.4	3.6	0	0	50
06:07.7	Cough	87.5	84.9	29.2	2.5	0	0	50
06:37.6	Uroflow start	44.7	44.9	23.7	-0.2	0	1.6	50
06:41.5	Peak flow	44.1	57.2	27.4	12.1	11.4	0.8	50
06:49.4	Stop infusion	52.8	53.4	27.1	1.6	31.9	2.4	50
07:15.8	Uroflow stop	44	52.1	24.7	-8	275	0	0

Table 3 shows the pressure-flow findings obtained from the urodynamic examination. The average flow rate (10.5 ml/sec) and the maximum flow rate (22.3 ml/sec) are within the normal range, suggesting no significant obstruction in the urinary tract. The acceleration (5.3 ml/sec/sec) is also within the normal range, indicating a normal bladder expulsion capability. The opening pressure (0.5 cmH_2_O) and closing pressure (1.6 cmH_2_O) are low, which might suggest a mild dysfunction of the internal urethral sphincter, consistent with signs of stress urinary incontinence (SUI). However, the pressure at peak flow (12.1 cmH_2_O) and the mean pressure (0.5 cmH_2_O) are within the normal range, indicating no significant obstruction or detrusor underactivity. These findings, combined with the results of the 1-hour pad test (32 g of leakage) and the OABSS (7 points), support the diagnosis of SUI and OAB in this patient. It is important to note that while these urodynamic findings provide valuable insight into the patient's condition, determining the optimal treatment plan requires a multifaceted perspective, including careful consideration of the patient's desires and values.

**Table 3:** Pressure-flow findings from urodynamic examination

Category	Value	Unit
Maximum flow	22.3	ml/sec
Average flow	20.5	ml/sec
Voiding time	19	mm:ss.S
Flow time	18.2	mm:ss.S
Time to max flow	3.0	mm:ss.S
Voided volume	273.8	ml
Flow at 2 seconds	16	ml/sec
Acceleration	6.3	ml/sec/sec
Pressure at peak flow	25	cmH_2_O
Flow at peak pressure	15	ml/sec
Peak pressure	23	cmH_2_O
Mean pressure	22	cmH_2_O
Opening pressure	2.0	cmH_2_O
Closing pressure	-9.2	cmH_2_O

Decision support utilizing graph theory at our institution

At our institution, we developed a network graph based on data from previous TVT and VEL treatments. This network graph, which was described in a preliminary study, incorporates various factors such as TVT (a), VEL (b), pelvic floor muscle training: PFMT (c), smoking habits (d), breast cancer development (e), age (f), marital status (g), parity (h), hypertension (i), diabetes (j), obesity (k), stroke (l), dyslipidemia (m), menopausal state (n), pelvic surgery (o), infertility treatment (p), spinal nerve issues (q), and the desire to have children (r). Changes (Δ) in the 1-hour pad test score (s), ICIQ score (t), and OABSS (u) from baseline to one year after treatment were also incorporated as variables. When constructing the network graph, direct correlations between TVT (a), VEL (b), and PFMT (c) were omitted from the analysis to minimize redundancy and prioritize the relationships among a broader range of variables.

The patients’ specific conditions were visually evaluated using the network graph, as illustrated below, and the decision between a) TVT or b) VEL as a supplementary treatment was made based on this assessment. The A* algorithm, a heuristic graph traversal algorithm, was employed to identify the shortest path connecting the patient's specific symptoms and characteristics in the network graph.

The patient's condition did not improve despite receiving instructions on pelvic floor muscle training (PFMT), and she aimed to enhance both the Δ1-hour pad test and ΔOABSS outcomes. She encountered difficulties related to her desire to conceive, diabetes, and hypertension.

The shortest path that includes b) VEL is in the following sequence.

Path: s: Δ1-hour pad test → c: PFMT → j: diabetes → i: hypertension → r: desire to have children → b: VEL → u: Δ OABSS.

The total distance of this path was 3.935202947 (arbitrary units).

The shortest path that includes a) TVT is in the following sequence.

Path: s: Δ1-hour pad test → c: PFMT → a: TVT → r: desire to have children → i: hypertension → j: diabetes → u: ΔOABSS.

The total distance of this path is 3.973495479 (arbitrary units).

**Figure 1:**
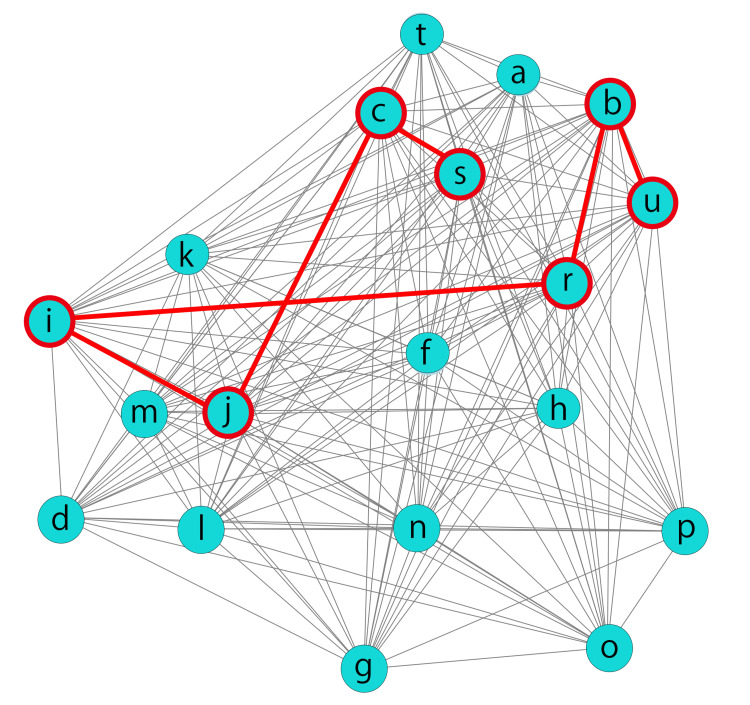
Optimizing treatment paths in a network graph In a network graph designed to support clinical decision-making, when a patient aiming to enhance both the 1-hour pad test improvement rate (s: Δ1-hour pad test) and OABSS improvement rate (u: ΔOABSS) presents with hypertension (i: hypertension), diabetes (j: diabetes), and a desire for childbearing (r: desire for children), the most efficient path to achieving these objectives is illustrated under the condition of ongoing pelvic floor muscle training (c: PFMT). Each variable in the network graph is denoted as follows. a: TVT; b: VEL; c: PFMT; d: smoking; e: breast cancer; f: age; g: marital status; h: parity; i: hypertension; j: diabetes; k: obesity; l: stroke; m: hyperlipidemia; n: menopausal status; o: pelvic surgery; p: infertility treatment; q: spinal nerve; r: desire for children; s: Δ1-hour pad test; t: ΔICIQ; and u: ΔOABSS. The lengths of the edges connecting the variables were inversely proportional to the correlation coefficients, with shorter edges indicating stronger relationships. The aggregate correlation coefficients for combinations a-b, a-c, and b-c were excluded from the analysis to prioritize the exploration of indirect relationships among the variables.

The network graph and the process of identifying the optimal treatment path based on the patient's specific conditions are visually represented in Figure 1.

Fotona laser treatment

**Figure 2:**
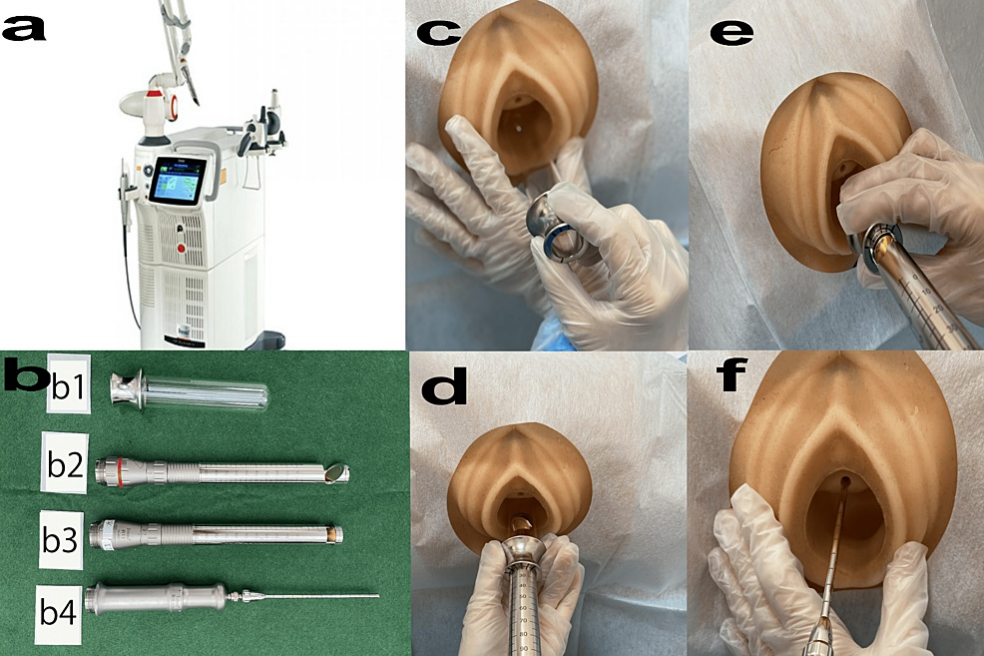
Fotona laser VEL + UEL treatment procedure a) SP Dynamis; Copyright © 2013. Provided courtesy of Fotona d.o.o. (Ljubljana, Slovenia). This image in Figure 2a is provided free of charge by Fotona d.o.o; b) b1, Special glass speculum for laser; b2, PS03 laser probe for the anterior wall of the vagina; b3, R11 laser probe for the entire circumference of the vagina; b4, R09-2Gu laser probe for the entire circumference of the urethra; c) VEL step (glass speculum insertion); d) VEL step (laser irradiation of anterior vaginal wall by PS03); e) VEL step (whole vaginal laser irradiation by R11); f) UEL step (whole urethral laser irradiation with R09-2Gu). VEL: vaginal erbium laser; UEL: urethral elevation lift

In the VEL + urethral extrusion length (UEL) treatment group, VEL was performed first, followed by UEL. During VEL, the vagina, labia, and urethra were disinfected with iodine and 8% xylocaine spray (Sandoz KK, Tokyo, Japan) was applied for 15 min to induce anesthesia. The SP Dynamis laser (Fotona d.o.o, Ljubljana, Slovenia) (Figure 2a) was used for laser irradiation, transitioning from VEL to UEL. Special glass vaginal specula and handpieces (Figure 2b, for PS03, R11, R09-2 Gu) were prepared for laser probes PS03, R11, and R09-2 Gu. Each handpiece was connected to the SP Dynamis laser.

First, a specially processed glass vaginal speculum capable of withstanding laser irradiation was inserted into the vagina (Figure 2c). The vaginal anterior wall was scanned using a PS03 laser probe with a spot size of 7 mm, pulse fluence of 6 J/cm², and a frequency of 2.0 Hz (Figure 2d). Laser irradiation was performed at 5 mm intervals, and this procedure was repeated three times.

Next, an R11 laser probe was used with a spot size of 7 mm, pulse fluence of 3.00 J/cm², and frequency of 2.0 Hz to perform laser treatment at 5 mm intervals over the entire 360-degree circumference of the vaginal canal (Figure 2e). This procedure was repeated twice.

A 10F Nelaton catheter was inserted through the urethra to drain residual urine from the bladder. The length of the urethra and sphincter were confirmed based on the relationship between the ultrasound and catheter position. Subsequently, the catheter was removed from the urethra, and the R09-2 Gu laser probe, designed for urethral use, was then inserted. The laser treatment settings were R09-2 Gu, SMOOTH, 1.4 Hz, and 1.5 J/cm². Four-stack irradiation was performed at 2.5 mm intervals from the urethral orifice to the proximal end (Figure 2f). This treatment was repeated four times.

VEL + UEL treatment was performed three times (L1, L2, and L3) at one-month intervals, and SUI was re-evaluated six months after the third VEL + UEL treatment (L3) (L-T0.5).

Treatment progress

The patient received an explanation of the treatment plan based on the guideline algorithm. In addition, a network graph was used to visually capture various systemic diseases and patient preferences. This approach piqued her interest in VEL. After receiving informed consent for VEL, the patient chose it as a treatment option. The network graph was used to supplement the guideline-compliant treatment plan, ensuring that the patient's individual needs and preferences were considered. The patient rated her previous experiences with informed consent from physicians and the current informed consent process using a network graph on a VAS scale (0: not at all good to 10: very good). The patients’ rationale for these ratings is presented in the Appendices (patient evaluation of informed consent).

VEL treatment was performed once a month for three months. One month after the last VEL treatment, the one-hour pad test decreased to 2 g, and the OABSS improved to 3 points. At this point, the high number of nighttime urinations was considered to be due to nocturnal polyuria, and amlodipine was changed to losartan. As a result, the nocturnal frequency disappeared, and the OABSS became 0 points. Six months after treatment, both SUI and OAB remained well-controlled. With the resolution of urinary incontinence, the patient was able to exercise and plan a temporary discontinuation of diabetes medications. Additionally, the improvement in urinary incontinence led to a significant reduction in genital infections, eliminating the need for sudden changes in diabetes medications.

## Discussion

In this case, the graph theory-based decision support tool was useful in selecting a treatment method tailored to the individual needs of the SUI patient [21]. This is particularly helpful in selecting a minimally invasive and effective treatment for relatively young patients with a desire for pregnancy and comorbidities [2,21]. VEL treatment was highly effective for both SUI and OAB and greatly improved the patient's QOL. Additionally, changing antihypertensive medication led to further improvements in OAB. The gold standard for stress urinary incontinence (UI) that is unresponsive to pelvic floor muscle training is MUS, represented by TVT and TOT [5,8-20]. MUS has a high success rate and abundant evidence for SUI; however, although the complication rate is low, pain may occur with TVT [7-20]. If many patients experience anxiety, it is an important issue, even if the complication rate is low, because healthcare should be patient-centered [2,21]. Moreover, if pain occurs after MUS treatment, removal of the polypropylene mesh is not easy, and the tissue may not return to its original state [7,11,12]. In recent years, VEL has emerged [7]. VEL has a shorter history than TVT, and there are few randomized controlled trials (RCTs) [7]. Comparative studies between TVT and VEL are scarce, including RCTs and retrospective studies [21]. With improvements in ethical standards, the approval of RCTs such as TVT and VEL has become difficult, and patient-centered informed consent should be obtained using observational data [21]. The effectiveness of VEL in SUI has been demonstrated [2].

RCTs in 2024 and 2018 revealed that VEL was superior to sham treatment in improving urinary incontinence symptoms, QOL, and sexual function [26,27]. A study in 2020 showed effectiveness for mild to moderate SUI, but it was limited to severe cases [28]. Research data are limited [12]. Okui et al. compared pelvic floor muscle training, pelvic floor muscle training + TVT, and pelvic floor muscle training + VEL and clarified the following points [2]: (1) in the pelvic floor muscle training + TVT group, OAB worsened compared to the pelvic floor muscle training group; (2) pelvic floor muscle training and VEL had a significant effect on OAB. This finding suggests that TVT surgery may not always benefit patients [2,29]. VEL showed equivalent effects to TVT in improving pad tests and International Consultation on Incontinence Questionnaire-Short Form (ICIQ-SF); however, VEL was superior to TVT in terms of OABSS [2,28,29]. Additionally, VEL tends to be selected for patients who desire pregnancy or wish to avoid foreign objects [2]. However, VEL requires periodic treatment continuation because of tissue aging [7].

When there is a large difference in evidence, the data have different dimensions [30,31]. Therefore, when providing information to patients, the TVT and VEL cannot be compared in the same dimension. This is known as discrete data [24]. Okui was the first to report the integration of discrete data into personalized medicine and named this the discrete mathematical approach [21,22]. In this case, the patient emphasized the keywords "hypertension," "diabetes," "desire for pregnancy," and "urinary incontinence." The physicians the patient encountered could not propose a comprehensive treatment. In contrast, the discrete mathematical approach efficiently finds a path that connects all keywords and explains that VEL is included in it [2]. In discrete mathematics, this concept is known as the traveling salesman problem (TSP) [30].

TSP has brought many benefits to modern society such as the development and refinement of optimization algorithms, the evolution of heuristics and approximation algorithms, and contributions to combinatorial optimization and theoretical computer science. Particularly famous applications include route optimization represented by Google Navigation [31] and genetic information analysis for DNA sequencing fragment assembly [32]. The graph theory-based decision support tool was useful in this case, but further verification of its validity and reproducibility is necessary. In particular, it is important to apply this tool to more cases and continuously improve it, especially in terms of variable selection and setting relationships between variables. Additionally, long-term follow-up is needed to verify the validity of the treatment choices using this tool and to accumulate evidence. Collecting patient feedback on informed consent used in this study is an important evaluation tool [33].

Finally, we consider the relationship with the global guidelines. The discrete mathematical approach using AI is not intended to replace existing guidelines but rather to complement them. Various guidelines emphasize the importance of gathering detailed information from patients and considering their individual circumstances when making treatment decisions. The Japanese guidelines highlight the importance of a comprehensive initial evaluation and emphasize the need to consider the patient's quality of life and preferences, assessing risk factors such as obesity/high BMI, aging, number of childbirths, smoking, hypertension, diabetes, alcohol consumption, and exercise [8]. The American Urological Association guidelines provide detailed steps for patient evaluation and counseling to ensure a holistic approach to treatment. Patients should be counseled on all available treatment options, including nonsurgical and surgical interventions, and potential complications specific to these options should be discussed [13]. The National Institute for Health and Care Excellence in the UK emphasizes the use of validated tools and comprehensive assessments. Clinicians should identify relevant predisposing factors and other diagnoses that may require referral using validated urinary incontinence-specific symptoms, quality-of-life questionnaires, and bladder diaries [15]. The European Association of Urology guidelines stress a holistic approach that incorporates both medical and psychological support. A detailed history and physical examination are essential, and addressing patients' concerns and expectations is crucial for improving the outcomes [16].

Despite the comprehensive nature of these guidelines, the patient's true desires and preferences are not always adequately reflected in their treatment plans. This highlights the need for AI to play a complementary role in supporting guideline execution. The discrete mathematical approach is one such idea that can enhance the integration of patient preferences into treatment plans. The patient's feedback on the informed consent process used in this study underscores the value of this approach in prioritizing the patient's feelings and preferences. By integrating AI-based decision support tools with existing guidelines, we can move toward a more patient-centered approach to healthcare, ensuring that individual needs and values are at the forefront of treatment decision-making.

## Conclusions

The graph theory-based decision support tool effectively optimized treatment selection for this 36-year-old woman with comorbid SUI, OAB, and desire for pregnancy. By analyzing her individual circumstances, the tool identified VEL treatment as the most suitable option. After VEL treatment, the patient experienced significant improvements in SUI and OAB symptoms, and a change in antihypertensive medication resolved nocturia, thus enhancing her quality of life. This case demonstrates the potential of the graph theory-based tool in providing personalized, data-driven treatment decision-making for SUI patients with complex needs. However, further research is necessary to validate the effectiveness and reproducibility of this tool in larger patient populations.

## Appendices

Patient's evaluation of informed consent

The patient rated Dr. A's explanation with a visual analog scale (VAS) score of 6 points (0 points being very unsatisfied and 10 points being very satisfied). The reason for this was that the recommendation of TVT surgery for SUI was appropriate, and it was understandable that TVT surgery is safe and has few sequelae based on long-standing documented evidence. However, the problem is that if the patient were to experience sequelae, such as worsening of OAB, she would have no choice but to accept it. If possible, she would like to avoid taking OAB medications, let alone SNM insertion, as she is young. Dr. A did not propose methods to avoid these risks. The doctor shifted the responsibility for hypertension and diabetes, which would likely increase the risk to the internist. Regarding reproduction, no data were shown and the topic was avoided.

The patient rated Dr. B's explanation with a VAS score of five points. It was appreciated that the doctor empathized with the keyword "reproduction" as an important issue. However, this doctor did not understand how much the patient's QOL was impaired by SUI and did not explore ways to proceed with SUI treatment while continuing reproductive medicine.

The patient rated Dr. C's explanation with a VAS score of five points. It was commendable that the doctor presented with the issue of developing cellulitis after TVT or TOT surgery. However, as a family physician who always manages the patient, the doctor should have organized the evidence and information to make it easier for the urologist to select surgical treatment based on various types of information. In addition, the doctor did not realize that nocturnal polyuria, which was thought to be a part of OAB, was actually caused by increased venous return at night due to amlodipine.

The patient rated Dr. D with a VAS score of 4. In the explanation of TOT surgery, ease compared to TVT surgery is a matter of the surgeon's skill and is not directly related to the patient. Moreover, no evidence has shown that a shorter mesh in TOT reduces the risk compared to TVT. Furthermore, the lack of a TVT option resulted in a greater reduction than that of Dr. A.

The patient provided informed consent using a network graph with a VAS score of 9 points. This was because detailed explanations of the VEL and TVT are appreciated. It was not a matter of which of VEL or TVT is superior in which situations, but rather, all the keywords "urinary incontinence," "overactive bladder," "desire for pregnancy," "hypertension," and "diabetes" were included in the same field of view, which earned a high rating. As a result, it was also determined that the cause of nocturnal polyuria was amlodipine. Unfortunately, a lack of long-term evidence is a point of deduction. Based on this informed consent, the patient thought as follows: "First, I will improve SUI with VEL while proceeding with treatment for hypertension and diabetes and achieving a successful pregnancy. Next, I will consider whether to perform TVT surgery. I think the risk factors will be reduced by then."
